# Searching for biological processes as targets for rheumatoid arthritis targeted therapy with ANDSystem, an integrated software and information platform

**DOI:** 10.18699/vjgb-25-107

**Published:** 2025-12

**Authors:** E.L. Mishchenko, I.V. Yatsyk, P.S. Demenkov, A.V. Adamovskaya, T.V. Ivanisenko, M.A. Kleshchev, V.A. Ivanisenko

**Affiliations:** Institute of Cytology and Genetics of the Siberian Branch of the Russian Academy of Sciences, Novosibirsk, Russia; Institute of Cytology and Genetics of the Siberian Branch of the Russian Academy of Sciences, Novosibirsk, Russia; Institute of Cytology and Genetics of the Siberian Branch of the Russian Academy of Sciences, Novosibirsk, Russia; Institute of Cytology and Genetics of the Siberian Branch of the Russian Academy of Sciences, Novosibirsk, Russia; Institute of Cytology and Genetics of the Siberian Branch of the Russian Academy of Sciences, Novosibirsk, Russia; Institute of Cytology and Genetics of the Siberian Branch of the Russian Academy of Sciences, Novosibirsk, Russia; Institute of Cytology and Genetics of the Siberian Branch of the Russian Academy of Sciences, Novosibirsk, Russia Novosibirsk State University, Novosibirsk, Russia

**Keywords:** rheumatoid arthritis, gene networks, targeted therapy, ANDSystem, ревматоидный артрит, генные сети, таргетная терапия, ANDSystem

## Abstract

Rheumatoid arthritis (RA) is a systemic autoimmune disease characterized primarily by joint involvement with progressive destruction of cartilage and bone tissue. To date, RA remains an incurable disease that leads to a significant deterioration in quality of life and patient disability. Despite a wide arsenal of disease-modifying antirheumatic drugs, approximately 40 % of patients show an insufficient response to standard treatment, highlighting the urgent need to identify new pharmacological targets. The aim of this study was to search for novel biological processes that could serve as promising targets for the targeted therapy of RA. To achieve this goal, we employed an approach based on the automated extraction of knowledge from scientific publications and biomedical databases using the ANDSystem software. This approach involved the reconstruction and subsequent analysis of two types of associative gene networks: a) gene networks describing genes and proteins associated with the development of RA, and b) gene networks describing genes and proteins involved in the functional responses to drugs used for the disease’s therapy. The analysis of the reconstructed networks identified 11 biological processes that play a significant role in the pathogenesis of RA but are not yet direct targets of existing disease-modifying antirheumatic drugs. The most promising of these, described by Gene Ontology terms, include: a) the Toll-like receptor signaling pathway; b) neutrophil activation; c) regulation of osteoblast differentiation; d) regulation of osteoclast differentiation; e) the prostaglandin biosynthetic process, and f) the canonical Wnt signaling pathway. The identified biological processes and their key regulators represent promising targets for the development of new drugs capable of improving the efficacy of RA therapy, particularly in patients resistant to existing treatments. The developed approach can also be successfully applied to the search for new targeted therapy targets for other diseases.

## Abstract (ru)

Ревматоидный артрит (РА) – системное аутоиммунное заболевание, сопровождающееся поражением преимущественно суставов с прогрессирующей деструкцией хрящевой и костной тканей. До настоящего времени РА остается неизлечимым заболеванием, приводящим к значительному ухудшению качества жизни и инвалидизации пациентов. Несмотря на наличие широкого арсенала базисных противовоспалительных препаратов, около 40 % пациентов демонстрируют недостаточный ответ на стандартное лечение, что подчеркивает острую необходимость поиска новых фармакологических мишеней. Целью настоящей работы был поиск новых биологических процессов, которые могут служить перспективными мишенями для таргетной терапии РА. Для достижения поставленной цели был применен подход, основанный на автоматическом извлечении знаний из текстов научных публикаций и биомедицинских баз данных с помощью программно-информационной системы ANDSystem. Данный подход включал реконструкцию и последующий анализ ассоциативных генных сетей двух типов: а) генные сети, описывающие гены и белки, ассоциированные с развитием РА, и б) генные сети, описывающие гены и белки, вовлеченные в функциональные ответы на действие лекарств, применяемых для терапии заболевания. В результате анализа реконструированных сетей выявлено 11 биологических процессов, играющих значимую роль в патогенезе ревматоидного артрита, но до сих пор не являющихся прямыми мишенями существующих базисных противовоспалительных препаратов. К числу наиболее перспективных относятся следующие процессы, описываемые терминами онтологии генов: а) сигнальный путь Toll-подобных рецепторов; б) активация нейтрофилов; в) регуляция дифференцировки остеобластов; г) регуляция дифференцировки остеокластов; д) биосинтез простагландинов; е) канонический сигнальный путь Wnt. Выявленные биологические процессы и их ключевые регуляторы представляют собой перспективные мишени для разработки новых лекарственных средств, способных повысить эффективность терапии РА, в том числе у пациентов, резистентных к существующим методам лечения. Разработанный подход может быть успешно использован для поиска новых мишеней таргетной терапии и при других заболеваниях.

## Introduction

Rheumatoid arthritis (RA) is a chronic autoimmune disease characterized by systemic inflammation that primarily affects the joints and leads to progressive destruction of cartilage and bone tissue (Guo et al., 2018). According to the World Health Organization, RA affects approximately 0.5–0.6 % of the global population, occurring 2–3 times more frequently in women than in men, and is one of the leading causes of disability among working-age adults (Kvien et al., 2006; GBD 2023).

The pathogenesis of RA involves complex interactions between genetic factors, immune dysregulation, and environmental triggers, resulting in the activation of proinflammatory cytokines, infiltration of immune cells into the synovial membrane of the joints, and chronic inflammation (Firestein, McInnes, 2017). Despite significant progress in understanding the molecular mechanisms of RA, complete remission of the disease remains unattainable, and current therapeutic strategies are primarily aimed at preventing disease progression (Smolen et al., 2016).

Modern treatment strategies for rheumatoid arthritis are based on the use of several classes of drugs with anti-inflammatory effects (Ding et al., 2023; Smolen et al., 2023), including: a) conventional synthetic (cs) disease-modifying antirheumatic drugs (csDMARDs) such as methotrexate, leflunomide, sulfasalazine, and hydroxychloroquine; b) targeted synthetic (ts) DMARDs (tsDMARDs) such as tofacitinib and baricitinib; c) biologic DMARDs (bDMARDs), including inhibitors of tumor necrosis factor (infliximab, adalimumab), interleukin-6 (tocilizumab, sarilumab), interleukin-1 (anakinra), and anti- CD20 monoclonal antibodies (rituximab); d) nonsteroidal anti-inflammatory drugs (NSAIDs) for symptomatic treatment; and e) glucocorticoids (GCs) for rapid suppression of inflammation.

Particular attention in clinical practice is given to first-line drugs such as csDMARDs and tsDMARDs, which are capable of modulating immune responses at the level of intracellular signaling pathways and metabolism (van der Kooij et al., 2007). The action of tsDMARDs, in particular, targets specific genes encoding key components of the JAK/STAT signaling pathway. For instance, tofacitinib suppresses inflammation by specifically inhibiting Janus kinase 3 (JAK3), which plays a crucial role in cytokine signaling that regulates lymphocyte survival, proliferation, differentiation, and apoptosis (Adis Editorial, 2010). Although csDMARDs and tsDMARDs are effective in achieving remission in a substantial proportion of patients, their use is limited by side effects such as hepatotoxicity, immunosuppression, and the development of resistance (Olivera et al., 2020). Moreover, approximately 40 % of RA patients exhibit a poor response to therapy, and 5–20 % show no improvement at all with standard treatment (Smolen et al., 2016), highlighting the need to identify new molecular targets for the development of more effective therapeutic agents.

The development of rheumatoid arthritis involves a number of signaling pathways – including JAK/STAT, Notch, MAPK, Wnt, PI3K, SYK, and others – which regulate many biological processes implicated in the pathogenesis of the disease, such as the inflammatory response and remodeling of bone and cartilage tissue (Ding et al., 2023). These and other biological processes and signaling pathways can serve as potential targets for RA drug therapy. For example, experiments in laboratory mice have shown that treatment with CEP-33779 – a highly selective inhibitor of JAK2, a key component of the JAK/STAT signaling pathway – can reduce inflammatory manifestations in arthritis by suppressing cytokine production and the activation of T and B lymphocytes (Stump et al., 2011).

The aim of our study was to identify biological processes – new promising pharmacological targets for rheumatoid arthritis therapy – based on the reconstruction and analysis of a specific type of gene network known as an associative gene network (AGN).

A gene network is a group of coordinately functioning genes that control the phenotypic traits of an organism (Kolchanov et al., 2013). Interactions between genes within a gene network occur through their primary and secondary products – RNAs, proteins, and metabolites. An associative gene network represents an extension of the traditional gene network, integrating genomic, molecular, phenotypic, and environmental entities and describing diverse types of interactions and associations among them (Demenkov et al., 2021).

To reconstruct AGNs, we used the ANDSystem software platform, which enables the automatic extraction of knowledge and facts from scientific publications and biomedical factual databases (Ivanisenko V.A. et al., 2019). To achieve this goal, the following tasks were addressed: a) reconstruction of an associative gene network for RA, including genes and proteins involved in the development of the disease; b) reconstruction of associative gene networks describing the mechanisms of action of drugs used in RA therapy, including genes and proteins participating in the functional response to these drugs; and c) identification, based on the reconstructed associative gene networks, of biological processes representing promising targets for RA therapy.

Based on the approach described above, 11 biological processes were identified that play a significant role in the development of rheumatoid arthritis but have not yet been recognized as direct targets of currently used disease-modifying antirheumatic drugs (DMARDs). These processes, described by Gene Ontology terms, include: a) the Toll-like receptor signaling pathway, b) neutrophil activation, c) regulation of osteoblast differentiation, d) regulation of osteoclast differentiation, e) prostaglandin biosynthetic process, and f ) the canonical Wnt signaling pathway. The identified biological processes and their key regulators represent promising targets for the development of new therapeutic agents for rheumatoid arthritis. The approach implemented in this study can also be applied to the identification of novel targets for targeted therapy in other diseases.

## Materials and methods

**List of disease-modifying antirheumatic drugs (DMARDs).** To compile a list of conventional synthetic DMARDs and targeted synthetic DMARDs used in the treatment of rheumatoid arthritis, we referred to the official document of the All- Russian Public Organization “Association of Rheumatologists of Russia” – “Clinical Guidelines: Rheumatoid Arthritis (ICD-10: M05, M06)” (Nasonov et al., 2024). This document provides a classification of drugs used for RA therapy, their pharmacotherapeutic characteristics, and Anatomical Therapeutic Chemical (ATC) classification codes. Based on these recommendations, the following list of drugs was compiled for further analysis: csDMARDs (methotrexate, leflunomide, sulfasalazine, hydroxychloroquine) and tsDMARDs (tofacitinib, baricitinib).

**Reconstruction and analysis of associative gene networks.**The reconstruction of associative gene networks was performed using the ANDSystem software and information platform (Ivanisenko V.A. et al., 2019, 2024; Ivanisenko T.V. et al., 2024). This system is based on methods of machine reading and artificial intelligence designed for the automatic extraction of knowledge and facts from large-scale genetic and biomedical data sources, such as scientific publications, patents, and factual databases.

Through the analysis of more than 40 million scientific articles and patents, as well as 150 factual databases, the ANDSystem knowledge base has accumulated biomedically significant information represented as semantic knowledge graphs, describing 12 types of biological entities (including genes, proteins, diseases, biological processes, drugs, etc.) and over 40 types of functional relationships among them. These relationships include gene expression regulation, protein degradation, modification, and transport, as well as physical interactions such as protein–protein and protein–ligand interactions.

In addition, the ANDSystem knowledge base contains descriptions of associative relationships linking genes, proteins, and metabolites with entities such as diseases, biological processes, and pharmaceutical compounds (Ivanisenko V.A. et al., 2019, 2024; Ivanisenko T.V. et al., 2024). The knowledge base also includes “marker” relationships, indicating that certain genes, proteins, biological processes, or phenotypic traits can serve as markers of specific diseases

**Identification of biological processes based on information from reconstructed associative gene networks.** The analysis of overrepresented biological processes in the reconstructed associative gene networks was carried out using the DAVID web server, version 2021 (https://david.ncifcrf.gov/; Sherman et al., 2022), with default settings. DAVID evaluates the degree of overlap between the list of genes functioning within each reconstructed gene network and the lists of genes corresponding to biological processes described in the Gene Ontology (GO). Based on this comparison, the hypergeometric test was applied to calculate the probability that the observed overlap between gene lists could occur by chance. In our study, biological processes significantly associated with the reconstructed gene networks were identified using a p-value threshold of <0.05, corrected by the Bonferroni method. The biological processes that met this criterion were classified into two categories: a) biological processes significant for the rheumatoid arthritis gene network, and b) biological processes significant for the gene networks representing responses to csDMARD and tsDMARD therapies used in RA treatment.

## Results

**Reconstruction of the associative gene network of rheumatoid arthritis**

Using the ANDSystem platform, we reconstructed the associative gene network of rheumatoid arthritis based on information contained in the ANDSystem knowledge base.

The graph of the reconstructed associative gene network had a star-shaped topology: the central node corresponding to the term “Rheumatoid arthritis” was connected by edges to other nodes of the network graph that represented proteins and genes associated with RA according to the ANDSystem knowledge base (Supplementary Fig. S1)1. In total, the graph contained 4,685 nodes, corresponding to 2,178 genes and 2,507 proteins (Table S1 in the Appendix), as well as 9,877 edges between the central node (rheumatoid arthritis) and the other nodes. Note that the number of edges exceeded the number of nodes. This is because the same node representing a gene or protein could be linked to the central node by multiple edges, each of which, according to the ANDSystem knowledge base, described a specific type of interaction between RA and a given gene or protein.


Supplementary Materials are available in the online version of the paper: https://vavilovj-icg.ru/download/pict-2025-29/appx37.xlsx


Table S1 lists the genes and proteins included in the reconstructed associative gene network of rheumatoid arthritis, which comprises, in particular, genes and proteins involved in the inflammatory process: interleukins (IL1, IL6, IL13, and others), members of the tumor necrosis factor (TNF) family, the key inflammatory regulator NF-κB, and genes and proteins functioning in the Wnt, JAK/STAT, Notch, MAPK, PI3K, and SYK signaling pathways, all of which are known to play a defining role in RA pathogenesis (Ding et al., 2024).

Table 1 presents a classification of 14 types of relationships between the central and peripheral nodes in the RA gene network. These relationships fall into two categories. The first category (regulatory relationships) comprises nine types, such as expression downregulation, expression upregulation, activity regulation, and others. For example, expression of interleukin-1 beta (IL1B) is increased in rheumatoid arthritis (Mohd et al., 2019), which is reflected in the ANDSystem knowledge base as an “expression upregulation” relationship between RA and the IL1B protein. Interleukin-6 (IL6) stimulates fibroblasts in the synovial membrane of the joints (Singh et al., 2021) and contributes to one of the symptoms of RA (bone loss), which is represented in ANDSystem as a “positive regulation” relationship between the disease “Rheumatoid arthritis” and the IL6 protein

**Table 1.**
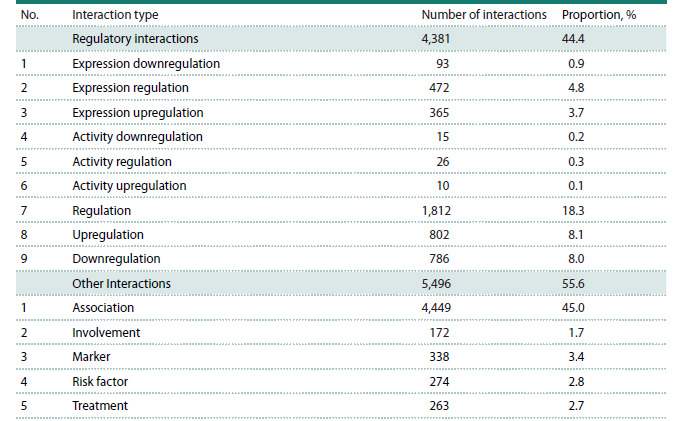
Characteristics of relationships between the central and peripheral nodes in the rheumatoid arthritis gene network * The percentage (%) indicates the proportion of a specific relationship type relative to the total number of relationships in the associative gene network of rheumatoid arthritis.

The second category (other relationships) includes five additional relationship types identified during the reconstruction of the RA gene network, describing situations in which a gene or protein is associated with RA in some way. For example, these may include structural or functional features of a gene if a mutation in that gene constitutes a risk factor for RA.

**Fig. 1.**
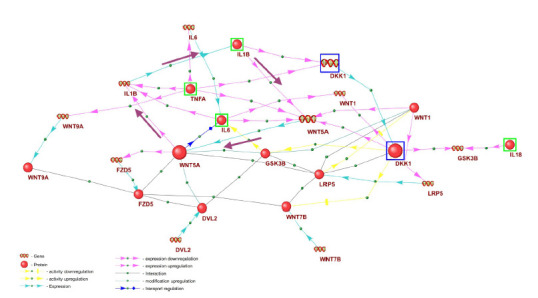
Mechanism of regulation of key components of the Wnt signaling pathway by proinflammatory cytokines, reconstructed from the rheumatoid arthritis gene network in the ANDSystem knowledge base Proinflammatory cytokines are highlighted with green frames; components of the positive feedback regulatory loop are indicated with bold arrows; and the DKK1 gene and its encoded protein Dickkopf-1 (DKK1) – an inhibitor of the canonical Wnt pathway – are shown in blue frames.

Based on the information contained in the associative gene network of rheumatoid arthritis and the ANDSystem knowledge base, it is possible to reconstruct the detailed mechanisms underlying the involvement of specific genes and proteins in the development of RA. Figure 1 illustrates, as an example, the regulatory interactions between genes and proteins functioning within the Wnt signaling pathway, which is regulated by proinflammatory cytokines such as interleukin-1 beta, tumor necrosis factor alpha (TNFA), and interleukin-6.

As shown in Figure 1, regulation of the Wnt signaling pathway in rheumatoid arthritis involves interleukin-1 beta, tumor necrosis factor alpha, and interleukin-6, which activate the expression of the WNT5A gene encoding the WNT5A protein – a ligand of FZD receptors participating in the noncanonical Wnt pathway (Miao et al., 2013). According to the ANDSystem data, WNT5A, in turn, activates the expression of the IL1B gene encoding interleukin-1 beta. Thus, IL1B and WNT5A mutually activate each other’s expression, forming a positive feedback loop, indicated in Figure 1 by bold arrows

**Reconstruction of associative gene networks involved in functional responses to RA therapies**

**Fig. 2.**
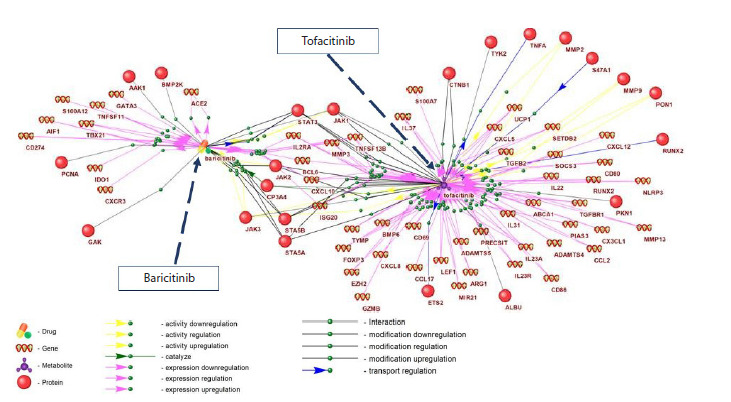
Reconstructed associative gene network of the response to two targeted synthetic disease-modifying antirheumatic drugs – tofacitinib and baricitinib

Figure 2 shows the AGN for responses to tsDMARDs (see also Table S2). It contains two nodes corresponding to the drug names (tofacitinib, baricitinib) and 157 edges linking these nodes to other nodes representing 22 proteins and 51 genes. As seen in Figure 2, according to the ANDSystem knowledge base, tofacitinib is characterized by a substantially larger number of interactions with proteins and genes (60) compared to baricitinib (26). In response to both drugs, genes involved in the inflammatory response – MMP3, IL2RA, CXCL10 – and proteins (STAT3, STAT5A, JAK1, JAK2), members of the JAK/STAT pathway, were implicated

Figure S2 presents the AGN for responses to csDMARDs (methotrexate, leflunomide, sulfasalazine, hydroxychloroquine). The graph contains 261 nodes, four of which correspond to the drug names (see also Table S2). The remaining nodes are connected to these four drug nodes by 485 edges and represent 106 proteins and 151 genes. The largest number of interactions in the csDMARD response AGN was observed for methotrexate (160). Proteins and genes associated with this drug include, in particular, IL1R1, TNFA, the inflammatory transcription factor NFKB1, and caspases (CASP1, CASP3, CASP9). Hydroxychloroquine ranked second by number of interactions (73), being linked to proinflammatory cytokines such as IL1B and TNFA, as well as to catalase (CAT) and cytochromes involved in xenobiotic metabolism (CP2B6, CYP1B1). Sulfasalazine and leflunomide ranked third and fourth (26 and 17 interactions, respectively). Notably, some proteins in the csDMARD response AGN (e. g., IL1B, CCL2, TNFA, CASP3) are targets of multiple drugs

The distribution of interaction types in the AGN of the response to csDMARDs and tsDMARDs is provided in Table 2. As can be seen from Table 2, regulatory interactions, particularly the regulation of gene expression, predominated among those in the AGN of the response to csDMARD and tsDMARD.

**Table 2.**
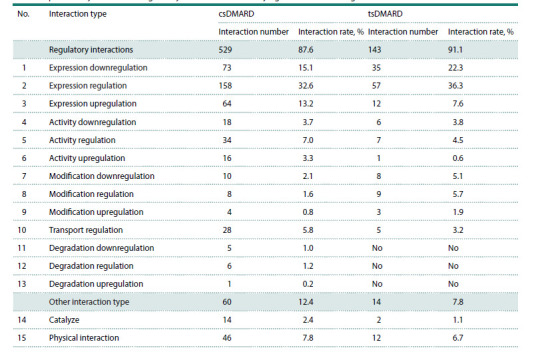
Distribution of interaction types in the reconstructed associative gene networks of the response to synthetic and targeted synthetic disease-modifying antirheumatic drugs

**Identification of biological processes based on information from reconstructed associative gene networks**

Using the DAVID web resource based on Gene Ontology, an overrepresentation analysis of biological processes in the reconstructed gene networks was performed for: a) the rheumatoid arthritis gene network and b) the gene networks of the response to two types of anti-inflammatory drugs (csDMARD and tsDMARD).

For the reconstructed associative gene networks of rheumatoid arthritis and the response to csDMARD and tsDMARD, 381, 64, and 44 overrepresented biological processes were identified, respectively. Most significant processes are characterized in Table 3 (for details, see Tables S4–S6). As seen in Table 3, the inflammatory response (GO identifier: GO:0006954) was statistically significantly overrepresented in both the RA gene network and the gene networks of the response to csDMARD and tsDMARD. It is interesting to note that the list of most significantly overrepresented processes for csDMARD response gene network included xenobiotic metabolic processes, which were not overrepresented in the tsDMARD gene network. For the tsDMARD response gene network, the JAK/STAT (GO identifier: GO:0007259, Table 3) and cytokine (GO identifier: GO:0019221, Table 3) signaling pathways were most significantly overrepresented.

**Table 3.**
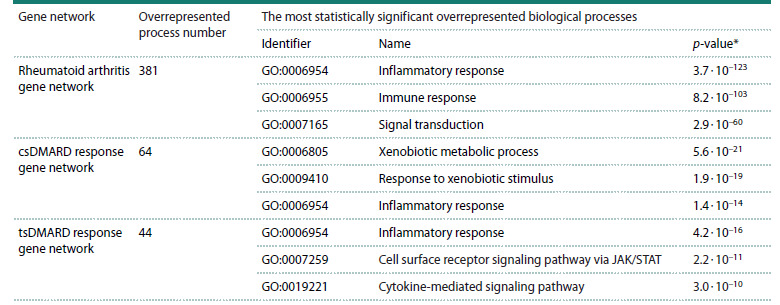
Results of the overrepresentation analysis of Gene Ontology (GO) biological processes for the associative gene networks of rheumatoid arthritis, as well as the gene networks of the response to synthetic disease-modifying antirheumatic drugs (сsDMARD) and targeted synthetic disease-modifying antirheumatic drugs (tsDMARD) * p <0.05.

For further analysis, from the 381 identified biological processes overrepresented in the RA AGN (Table 3), 71 processes were selected using the ANDSystem knowledge base, characterized by the interaction types “Regulation”, “Downregulation”, and “Upregulation” with the disease “Rheumatoid arthritis”. An intersection was performed between the list of 71 biological processes involved in the pathogenesis of RA and the lists of overrepresented biological processes for the AGN of the response to the csDMARD (64 processes) and tsDMARD (44 processes) drug groups. As a result, 59 biological processes were found that are involved in the pathogenesis of RA but are not included in the list of overrepresented processes for the AGN of the response to the considered drugs. From these 59 processes, 48 were removed that, according to the ANDSystem knowledge base, are linked to the considered csDMARD (methotrexate, leflunomide, sulfasalazine, hydroxychloroquine) and tsDMARD (tofacitinib, baricitinib) drugs by interactions of the types “Regulation”, “Downregulation”, and “Upregulation”.

This resulted in a list of 11 biological processes (Table 4). The identified processes are characterized by the following: firstly, these processes are involved in the pathogenesis of rheumatoid arthritis. Furthermore, no regulating csDMARDs and tsDMARDs have been identified for them. It is these processes that are of particular interest as targets for the development of drugs for rheumatoid arthritis therapy.

**Table 4.**
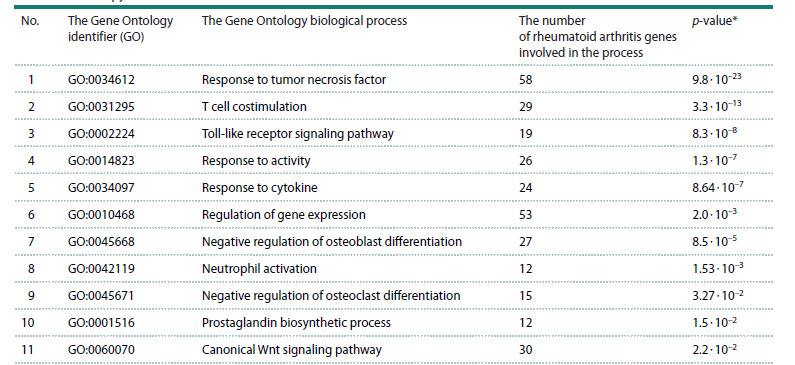
Biological processes for which no regulating drugs from the csDMARD and tsDMARD groups used in the therapy of rheumatoid arthritis have been identified * p-value – significance level of the overrepresentation of Gene Ontology terms for the set of genes associated with rheumatoid arthritis, with the Bonferroni correction.

As seen from Table 4, the biological processes involved in the pathogenesis of rheumatoid arthritis but not regulated by disease-modifying antirheumatic drugs included: a) inflammatory responses (GO identifiers GO:0034097, GO:0034612, GO:0031295, GO:0002224); b) bone tissue morphogenesis (GO:0045668, GO:0045671); c) the canonical Wnt signaling pathway (GO:0060070); d) prostaglandin biosynthesis (GO:0001516); e) response to activity (GO:0014823) and regulation of gene expression (GO:0010468).

Thus, we have conducted a search for biological processes – new promising pharmacological targets for RA therapy – based on the reconstruction and analysis of associative gene networks.

**Fig. 3.**
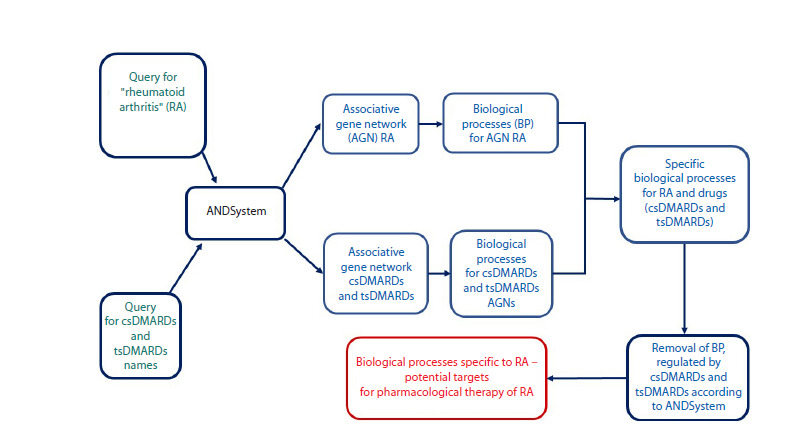
Main stages for searching for biological processes promising as targets for the development of new antirheumatic drugs. RА – rheumatoid arthritis; AGN – associative gene network; BP – biological process; csDMARD – conventional synthetic disease-modifying antirheumatic drugs (methotrexate, leflunomide, sulfasalazine, hydroxychloroquine); tsDMARD – targeted synthetic disease-modifying antirheumatic drugs (tofacitinib, baricitinib).

Figure 3 shows the schematic diagram, implemented in our work, for searching for biological processes that are new promising targets for the development of antirheumatic drugs.

## Discussion

The search for new drug targets for the treatment of rheumatoid arthritis is important for modern medicine, given that up to 40 % of patients do not achieve a full response to existing therapy (Ding et al., 2023). In this regard, we have proposed a method for identifying biological processes as targets for new antirheumatic drugs, based on the reconstruction of associative gene networks and a comparative analysis of biological processes associated with rheumatoid arthritis and those regulated by the disease-modifying antirheumatic drugs currently used in clinical practice (Nasonov et al., 2024).

The ANDSystem knowledge base, which we used for reconstructing the gene networks, integrates accumulated information from scientific literature on the molecular mechanisms of drug action and disease pathogenesis, allowing for the discovery of new therapeutic targets at a systemic level, including biological processes, thereby increasing the efficacy of therapy and diagnostics. In our work, we reconstructed associative gene networks (AGNs) for rheumatoid arthritis, as well as AGNs describing the interactions of synthetic and targeted anti-inflammatory drugs with human genes and proteins. The analysis showed that the rheumatoid arthritis gene network is enriched with genes involved in the regulation of the inflammatory response, which corresponds to the wellknown data on the leading role of systemic inflammation in the pathogenesis of this disease (Firestein, McInnes, 2017; Figus et al., 2021). It is therefore no coincidence that the reconstructed gene networks of proteins and genes targeted by csDMARDs (Fig. S2) and tsDMARDs (Fig. 2) primarily include genes and proteins involved in the functioning of the immune system.

According to the results of the functional annotation of genes, for conventional synthetic disease-modifying antirheumatic drugs, the list of statistically significantly overrepresented biological processes included processes related not only to inflammation but also to xenobiotic metabolism. This suggests that csDMARDs impose a significant load on the biochemical systems responsible for xenobiotic removal, potentially leading to serious adverse effects (Olivera et al., 2020).

On the other hand, for genes involved in the response to targeted synthetic disease-modifying antirheumatic drugs, xenobiotic metabolism processes were not significantly overrepresented. However, the list of overrepresented processes for tsDMARDs response gene network, along with inflammation, included processes related to the functioning of the JAK/ STAT signaling pathway, which is crucial for pathogenesis of RA (Ding et al., 2023). This suggests a more targeted action of tsDMARD on the pathogenesis of RA and emphasizes the importance of developing targeted therapies to increase treatment efficacy and reduce side effects. However, the diversity and complexity of the interactions of biological processes leading to the development of RA, and the insufficient efficacy of therapy with existing disease-modifying antirheumatic drugs, necessitate the search for new targets for RA treatment (Smolen et al., 2016).

Our approach, based on the reconstruction of gene networks involved in the development of the disease and in the response to known drugs, as well as on a comparative analysis of the biological processes regulated by these gene networks, allowed us to identify 11 biological processes (Table 4). These processes are key to the pathogenesis of RA but are not targets of the anti-inflammatory drugs currently in use. It should be noted that the regulation of expression (GO:0010468) and the response to activity (GO:0014823) belong to a group of rather broad processes, covering many molecular mechanisms in the cell, which complicates the development of targeted drugs.

Literature analysis revealed that for processes such as the response to cytokines (GO:0034097), the response to tumor necrosis factor TNFA (GO:0034612), and T-cell co-stimulation (GO:0031295), there is evidence of their partial regulation by the currently used csDMARDs and tsDMARDs. For example, tsDMARDs like tofacitinib and baricitinib effectively block the JAK/STAT signaling pathways, which are downstream of cytokine and TNFA receptors, providing powerful suppression of inflammatory responses (Palmroth et al., 2021).However, biological processes such as the Toll-like receptor signaling pathway, neutrophil activation, negative regulation of osteoblast differentiation, negative regulation of osteoclast differentiation, the canonical Wnt signaling pathway, and prostaglandin biosynthesis are not directly regulated by the disease-modifying antirheumatic drugs that are currently actively used by rheumatologists in accordance with clinical guidelines (Nasonov et al., 2024). Nevertheless, the biological processes and pathways listed above may be important for the pathogenesis of RA. For example, neutrophil activation plays an important role in inflammation in RA patients, and CXCR2 inhibitors, being investigated for other inflammatory conditions, could be adapted for RA (Alam et al., 2020).

It is known that the Wnt signaling pathway plays a significant role in fibroblast activation and synovial inflammation, as well as in bone resorption and joint destruction in the development of rheumatoid arthritis (Miao et al., 2013). The expression of genes encoding Wnt family proteins, which activate the Wnt signaling pathway, was increased in the synovium in rheumatoid arthritis, partly due to proinflammatory cytokines (Prajapati, Doshi et al., 2023). At the same time, the activation of the non-canonical Wnt signaling pathway, in turn, leads to an increased expression of inflammatory mediators, including the transcription factor NF-κB and cytokines (Miao et al., 2013), increasing inflammation

According to the ANDSystem knowledge base (Fig. 1), interleukin-1 beta and the WNT5A protein mutually activate each other’s expression, which may create a vicious cycle in the pathogenesis of rheumatoid arthritis. Therefore, modulating the Wnt signaling pathway may be a promising approach to reduce joint inflammation in RA. In particular, it has been shown that the NAV2 protein promotes the inflammatory response of fibrocyte-like synoviocytes by activating the Wnt signaling pathway in rheumatoid arthritis, and its inhibition can reduce joint inflammation in this disease (Wang R. et al., 2021).

On the other hand, proinflammatory cytokines – tumor necrosis factor-alpha and IL1B – according to ANDSystem (Fig. 2), can activate the expression of the DKK1 gene, which encodes the Dickkopf-1 (DKK1) protein, an important inhibitor of the canonical Wnt signaling pathway (Rabelo et al., 2010). It has been shown that the serum level of DKK1 is elevated in patients with RA and correlates with the level of inflammation and the degree of bone destruction in the joints (Wang S.Y. et al., 2011). The activation of DKK1 expression by proinflammatory cytokines in rheumatoid arthritis may lead to the suppression of the Wnt signaling pathway and, consequently, the activation of the RANK/RANKL signaling pathway in osteoclasts, increasing their activity and causing the bone loss characteristic of RA (Miao et al., 2013).

Thus, dysregulation of the Wnt signaling pathway may be the cause of changes in the biological processes of regulating osteoblast and osteoclast differentiation in RA, which, according to our study (Table 4), are potential targets for new antirheumatic drugs. Furthermore, DKK1 stimulates angiogenesis in the synovium and the formation of pannus – a pathologically altered synovial tissue that plays a crucial role in joint destruction in RA (Cici et al., 2019).

Thus, the Wnt signaling pathway is a promising target for the development of new antirheumatic drugs; however, its regulation in RA is very complex and depends on the type of tissues and cells, so further research is needed to reconstruct the gene network of this pathway in RA and analyze its structural and functional features in various cells and tissues.

Prostaglandins, particularly prostaglandin E2, are known to play an important role in the development of both acute inflammatory reactions and chronic inflammation (Kawahara et al., 2015), enhancing inflammatory processes by activating the expression of cytokine receptors and NFKB family proteins, which are key triggers of inflammation (Yao, Narumiya, 2019). Prostaglandin E2, an important mediator of inflammation in RA, is a target for a number of non-steroidal anti-inflammatory drugs (NSAIDs) for this disease (Park et al., 2006). The biosynthesis of prostaglandins (GO biological process identifier GO:0001516) is partially modulated by NSAIDs, such as celecoxib, but the development of more specific inhibitors could improve therapeutic outcomes (Gong et al., 2012).

It is known that toll-like receptors (TLRs) make an important contribution to the induction of inflammation, as their activation leads to increased activity of signaling pathways and a number of transcription factors such as nuclear factor κB (NF-κB), activator protein-1 (AP-1), and interferon regulatory factors (IRF), which induce the expression of proinflammatory cytokines – TNF, IL1β, IL6, and others (Kawasaki, Kawai, 2024). It has been shown that the expression of toll-like receptor genes is increased in the synovium of RA patients, and TLRs contribute significantly to the development of inflammation in RA, but therapeutic interventions targeting TLR signaling pathways have not yet been successfully introduced into clinical practice (Unterberger et al., 2021).

Thus, all the biological processes listed above play a major role in the development of RA, yet they are not regulated by the disease-modifying antirheumatic drugs currently used in clinical practice. Therefore, these biological processes and their key regulators can serve as targets for the development of new drugs for the treatment of rheumatoid arthritis.

It should be noted that rheumatoid arthritis is characterized by significant comorbidity with other diseases, including cardiovascular and respiratory diseases (Figus et al., 2021), anxiety-depressive disorders (Hill et al., 2022), and osteoporosis (Llorente et al., 2020). In this regard, further work is planned to analyze the identified biological processes as a basis for the comorbidity of RA with other diseases

Furthermore, this work did not identify targets at the gene level, which could be the subject of further research based on the analysis of the structural organization of gene networks

## Conclusion

In our work, we performed a computational reconstruction of associative gene networks for rheumatoid arthritis, as well as AGNs describing the interactions of synthetic and targeted anti-inflammatory drugs with human genes and proteins. Based on the analysis of these gene networks, a search for biological processes as new promising pharmacological targets for RA therapy was conducted. The proposed approach can also be used to search for new targets for therapy of other diseases where standard treatment methods show insufficient therapeutic effect.

## Conflict of interest

The authors declare no conflict of interest.
